# Efficacy and Safety of Sitagliptin Added to Insulin in Japanese Patients with Type 2 Diabetes: The EDIT Randomized Trial

**DOI:** 10.1371/journal.pone.0121988

**Published:** 2015-03-27

**Authors:** Seiji Sato, Yoshifumi Saisho, Kinsei Kou, Shu Meguro, Masami Tanaka, Junichiro Irie, Toshihide Kawai, Hiroshi Itoh

**Affiliations:** Department of Internal Medicine, Keio University School of Medicine, Tokyo, Japan; Weill Cornell Medical College Qatar, QATAR

## Abstract

**Aims:**

To clarify the efficacy and safety of adding sitagliptin to insulin therapy in Japanese patients with suboptimally controlled type 2 diabetes (T2DM).

**Study Design and Methods:**

This was a 24-week, prospective, randomized, open-labeled, controlled trial. Patients with T2DM who were suboptimally controlled despite receiving at least twice daily injection of insulin were enrolled in the study. The patients were randomized to continuation of insulin treatment (Insulin group) or addition of sitagliptin 50 to 100 mg daily to insulin treatment (Ins+Sita group). The primary outcome was change in HbA1c at week 24.

**Results:**

Adding sitagliptin to insulin significantly reduced HbA1c from 7.9 ± 1.0% at baseline to 7.0 ± 0.8% at week 24 (P <0.0001), while there was no significant change in HbA1c in the Insulin group (7.8 ± 0.7% vs. 7.8 ± 1.1%, P = 0.32). The difference in HbA1c reduction between the groups was 0.9% (95% confidence interval, 0.4 to 1.5, P = 0.01). There was no significant weight gain in either group. Incidence of hypoglycemia was significantly reduced in the Ins+Sita group compared with the Insulin group. Treatment satisfaction was improved in the Ins+Sita group. Baseline HbA1c level and beta cell function were associated with the magnitude of reduction in HbA1c in the Ins+Sita group.

**Conclusion:**

Adding sitagliptin to insulin reduced HbA1c without weight gain or increase in hypoglycemia, and improved treatment satisfaction in Japanese patients with T2DM who were suboptimally controlled despite at least twice daily injection of insulin.

**Trial Registration:**

The University Hospital Medical Information Network (UMIN) Clinical Trials Registry UMIN000004678

## Introduction

Type 2 diabetes (T2DM) is characterized by beta cell dysfunction and insulin resistance[1]. It is a progressive disease, and most patients with T2DM eventually require insulin therapy to achieve optimal glycemic control[2]. Insulin is the most effective glucose-lowering agent; however, since increased risk of hypoglycemia, weight gain, and fear or unwillingness to inject limits optimization of the dose and number of insulin injections, many patients treated with insulin still do not achieve their glycemic goal[2–4].

Dipeptidyl peptidase-4 (DPP-4) inhibitors slow the degradation of incretin hormones, glucagon-like peptide-1 (GLP-1) and glucose-dependent insulinotropic polypeptide (GIP), and thereby enhance the action of endogenous incretin[5]. Since incretin hormones stimulate insulin secretion in a glucose-dependent manner, DPP-4 inhibitors improve hyperglycemia without an increase in risk of hypoglycemia and weight gain[6]. DPP-4 inhibitors have also been shown to improve glucagon dynamics[7]. Furthermore, animal studies suggest that chronic exposure to GLP-1 may increase beta cell mass by promoting proliferation and differentiation and inhibiting apoptosis of beta cells[8].

Based on these characteristics of DPP-4 inhibitors, adding a DPP-4 inhibitor to insulin is expected to improve glycemic control without an increase in risk of hypoglycemia and weight gain. Previous studies conducted in USA and Europe have shown that adding a DPP-4 inhibitor to insulin in patients with T2DM reduced HbA1c[9–11], but the incidence of hypoglycemia was increased in one study in which sitagliptin was used[9]. Moreover, since the glucose-lowering effect of DPP-4 inhibitors appears to be greater in Asians compared with Caucasians[12], the efficacy and safety of DPP-4 inhibitors added to insulin need to be clarified in the Asian population as well as in other ethnic groups.

In Japan, the first DPP-4 inhibitor, sitagliptin, has been marketed since 2009. Therefore, in this study we aimed to evaluate the efficacy and safety of adding sitagliptin in Japanese patients with T2DM whose glycemic control is suboptimal despite insulin therapy.

## Research Design and Methods

### Subjects

**Fig 1:**
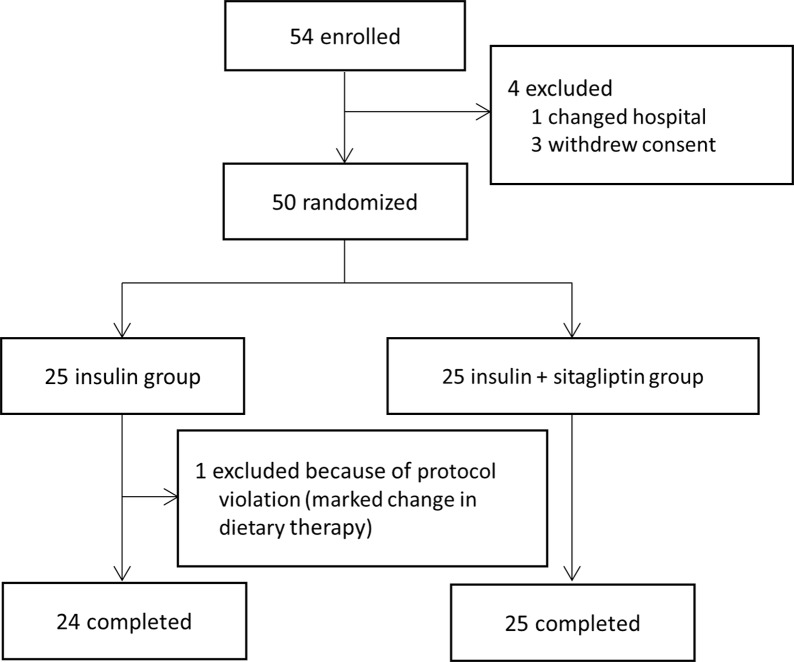
Flowchart of study participants.

The protocol for this trial and supporting CONSORT checklist are available as supporting information; see S1 CONSORT Checklist, S1 and S2 Protocol. We enrolled outpatients with T2DM who had been treated with at least twice daily injections of insulin for at least 2 months, and who had a suboptimal level of glycated hemoglobin (HbA1c) (i.e., ≥7.0%, according to the guidelines of the Japan Diabetes Society (JDS)[13]) between January 2011 and March 2013. A total of 54 patients were enrolled in the study, but four patients withdrew their consent before randomization (Fig. 1). No patients had been treated with sitagliptin or other DPP-4 inhibitors prior to study entry, since combination therapy with sitagliptin, the first approved DPP-4 inhibitor in Japan, and insulin was not approved in Japan until September 2011. Patients with type 1 diabetes, patients who had changed oral hypoglycemic agents within 2 months prior to study entry, and patients with severe liver disease or renal failure were excluded from the study. Written informed consent was obtained from all patients. This study was conducted according to the principles expressed in the Declaration of Helsinki and was approved by the ethical review committee of Keio University School of Medicine, Tokyo, Japan. The study was registered in the University Hospital Medical Information Network (UMIN) Clinical Trials Registry (http://www.umin.ac.jp/ctr/) as “Efficacy and safety of DPP-4 inhibitor added to Insulin treatment in patients with type 2 diabetes (EDIT) study; UMIN000004678”.

### Study design

This was a 24-week, prospective, randomized, open-labeled, controlled trial. A total of 50 subjects were randomized to the insulin (Insulin) group or the insulin plus sitagliptin (Ins+Sita) group based on a table of random numbers (simple randomization), and the treatment allocation was blinded to both attending physicians and patients until day 0 of the study. Subjects in the Insulin group continued their previous treatment during the study, and the insulin dose was adjusted by the attending physicians at each visit according to the guidelines of the Japan Diabetes Society (JDS) to achieve HbA1c below 7.0% as well as fasting plasma glucose levels below 130 mg/dL and 2 h postprandial plasma glucose level below 180 mg/dL[13]. In subjects in the Ins+Sita group, sitagliptin 50 mg once daily was added to the previous treatment at week 0, and if the patient randomized to the Ins+Sita group was treated with a sulfonylurea or glinide, these drugs were discontinued at week 0. Further, insulin dose, regardless of the type of insulin, was decreased by 20% at week 0 if the patient’s HbA1c was less than 8.0% at week 0. Then, the insulin dose was adjusted by the attending physicians at each visit during the study according to the JDS guidelines[13]. Adherence to sitagliptin treatment (75% or greater administration) was confirmed at each visit by assessing the medication possession ratio (%MPR; 99.1 ± 2.7%). If HbA1c did not achieve a level below 7.0% at week 12, the attending physicians were able to uptitrate the dose of sitagliptin to 100 mg daily. Other treatment including lifestyle modification was continued in both groups during the study. Patients in both groups were asked to visit the clinic every 4 weeks and received standard care similarly, except that patients allocated to the Ins+Sita group were also asked to attend for an additional visit at week 2, when the attending physicians judged whether further reduction in insulin was needed. The primary outcome of the study was change in HbA1c at week 24.

Blood and urine sampling was conducted at weeks 0, 4, 8, 12, 16, 20 and 24, together with body weight and blood pressure measurement. The patients were allowed to continue their self-monitoring of blood glucose (SMBG) during the study and instructed to measure glucose level if they felt hypoglycemic. In addition, the patients were asked to conduct 7-point SMBG at week 0 and 24 to assess the daily glycemic profile. Seven-point SMBG was conducted seven times a day, before and 1 h after three meals and at bedtime, for two consecutive days, and mean values were used for analysis. The incidence and severity of hypoglycemia were assessed by a questionnaire in the patients at each visit. Hypoglycemia was defined as having hypoglycemic symptoms and/or blood glucose less than 70 mg/dL. Severe hypoglycemia was defined as hypoglycemia requiring the assistance of a third party for recovery. The patients were also asked to answer the Diabetes Treatment Satisfaction Questionnaire (DTSQ)[14] and the Diabetes Medication Satisfaction Questionnaire (DiabMedSat)[15] to assess treatment satisfaction at weeks 0 and 24.

### Meal tolerance test

A mixed meal tolerance test (MTT) was administered at weeks 0 and 24. On the day of MTT, patients were asked to attend the hospital after an overnight fast and instructed to consume a meal within 15 min. The meal consisted of crackers, pudding and bisque soup (460 kcal; 56.5 g carbohydrate, 18 g fat and 18 g protein, Janefu E460F18, Kewpie Corporation, Tokyo, Japan). During MTT, the patients were asked to take medication as usual, except that the insulin dose was reduced by 50% to prevent hypoglycemia. Blood sampling was conducted at 0, 30, 60, 90 and 120 min after ingestion of the meal.

### Laboratory measurements

All measurements, unless otherwise indicated, were performed by the Department of Laboratory Medicine, Keio University School of Medicine using routine automated laboratory methods as previously described[16, 17]. HbA1c was measured by high-performance liquid chromatography (HPLC), and the values were expressed as National Glycohemoglobin Standardization Program (NGSP) values in this paper[18]. Serum glycated albumin (GA) and 1,5-anhydroglucitol (1,5-AG) were measured by enzymatic methods. Serum C-peptide immunoreactivity (CPR) was measured by chemiluminescent enzyme immunoassay (CLIA). Plasma intact proinsulin was measured by CLIA (Invitron Limited, Monmouth, UK), and the intact proinsulin (pmol/L)/CPR (pmol/L) ratio was calculated. CPR index was calculated as CPR (ng/mL) /glucose (mg/dL) x 100, and the area under the curve (AUC) of CPR to the AUC of glucose ratio was expressed as ng/mg. Glucagon was measured by radioimmunoassay (RIA) (Millipore Corporation, Billerica, MA, USA). Total GLP-1 was measured by electro-chemiluminescent immunoassay (Meso Scale Discovery, Gaithersburg, MD, USA), and intact GLP-1 and total GIP were measured by ELISA (Millipore), as previously reported[19]. Urinary 8-hydroxydeoxyguanosine (8-OHdG) was measured by ELISA (Japan Institute for the Control of Aging, Nikken Seil Co., Ltd., Shizuoka, Japan). Urinary 8-iso-prostaglandin F2alpha (8-iso-PGF2α) was measured by EIA (Cayman Chemical Company, Ann Arbor, MI, USA). Total and intact GLP-1, total GIP and proinsulin were measured at 0 and 60 min during MTT. Urinary excretion rates of 8-OHdG and 8-iso-PGF2α were assessed at week 0 and 24. Estimated glomerular filtration rate (eGFR: mL/min/1.73 m^2^) was calculated according to the Statement of the Japan Nephrology Society (JNS) as follows: 194 x serum creatinine (mg/dL)^-1.094^ x age (years)^-0.287^ (x 0.739 for women)[20].

### Statistical analysis

The study was powered to show superiority in the primary endpoint, HbA1c, at week 24. In order to detect a difference in HbA1c of 0.6%[9] with SD of 0.7% between the two treatment groups, 23 subjects per group would yield a power of 80% with a 5% two-sided significance level. Assuming a withdrawal rate of 10%, enrollment of 25 subjects per group was planned.

Data are presented as mean ± SD in the text and tables, and as mean ± 1.96 x standard error (SE) (i.e., 95% confidence interval; CI) in the figures. Non-normally distributed data are expressed as median (interquartile range; IQR). Statistical analyses were performed using SPSS version 22 (IBM, Chicago, IL, USA). Mann-Whitney U test or Fisher’s exact test was used to analyze the difference between the groups. The change in each parameter from baseline was analyzed by Wilcoxon signed-rank test. Spearman’s rank correlation coefficient was used to test the relationship between change in HbA1c and clinical parameters. For analysis of sensitivity, the longitudinal profile was also analyzed by mixed model repeated measures (MMRM). A P-value <0.05 was considered statistically significant.

## Results

### Patients’ characteristics

One patient in the Insulin group was excluded because of protocol violation (Fig. 1). Eventually, 49 patients (24 in Insulin group, 25 in Ins+Sita group) completed the study and were included in the analysis (Table 1). There was no significant difference in sex, age, duration of diabetes, BMI and insulin dose between the two groups (all P >0.05).

**Table 1:** Baseline characteristics of patients.

	Insulin group N = 24	Ins+Sita group N = 25	*P*
Male/female	18/6	16/9	0.40
Age (years)	66 ± 13	66 ± 8	0.37
Duration of diabetes mellitus (years)	20 ± 9	19 ± 8	0.90
Duration of insulin therapy (years)	7 ± 4	8 ± 3	0.40
Height (cm)	162 ± 9	165 ± 10	0.33
Body weight (kg)	70.6 ± 14.5	66.2 ± 9.9	0.47
BMI (kg/m^2^)	26.8 ± 4.3	24.5 ± 2.0	0.09
Systolic blood pressure (mmHg)	135 ± 21	132 ± 13	0.66
Diastolic blood pressure (mmHg)	75 ± 15	76 ± 10	0.32
Waist circumference (cm)	94.3 ± 9.2	89.2 ± 6.7	0.07
HbA1c (%)	7.8 ± 0.7	7.9 ± 1.0	0.94
Total cholesterol (mg/dL)	178 ± 26	188 ± 32	0.27
LDL cholesterol (mg/dL)	98 ± 26	99 ± 25	0.77
HDL cholesterol (mg/dL)	46 (42–58)	46 (39–59)	0.52
Triglycerides (mg/dL)	120 (93–188)	101 (60–136)	0.09
eGFR (mg/dL)	63.9 ± 16.7	64.0 ± 19.7	0.93
Diabetic retinopathy (%)	67	40	0.06
Diabetic nephropathy (%)	50	44	0.67
Diabetic neuropathy (%)	71	56	0.28
Macroangiopathy (%)	46	56	0.48
Insulin dose (U/kg/day)	0.46 ± 0.22	0.48 ± 0.26	0.35
Number of insulin injections (times/day)	3 ± 1	3 ± 1	0.42
Basal-bolus insulin therapy (%)	37.5	44	0.64
Premixed insulin (%)	62.5	56	0.64
Sulfonylurea (%)	8	4	0.53
Glinide (%)	8	12	0.67
Biguanide (%)	33	28	0.69
Thiazolidinedione (%)	17	4	0.14
α-glucosidase inhibitor (%)	42	36	0.68

BMI; body mass index, HbA1c; glycated hemoglobin, eGFR; estimated glomerular filtration rate, LDL; low-density lipoprotein, HDL; high-density lipoprotein. Data are expressed as mean ± SD or median (interquartile range).

### Glycemic control

**Fig 2:**
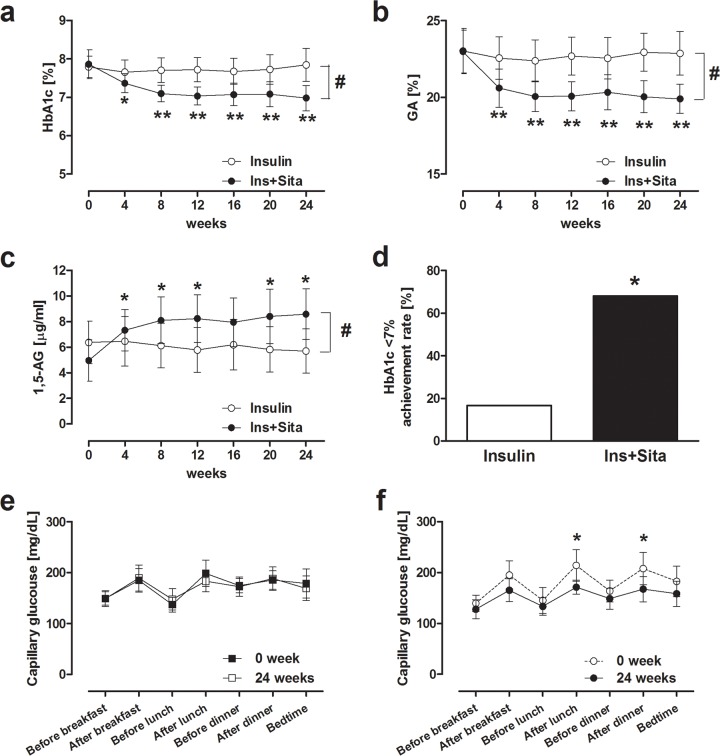
Changes in HbA1c (a), GA (b), 1,5-AG (c) and HbA1c <7% achievement rate (d) during the study. Changes in daily glycemic profile (7 point SMBG; before and after each meal and at bedtime) in insulin group (e) and insulin plus sitagliptin group (f). * *P* <0.05 and ** *P* <0.01 vs. baseline. # *P* <0.05 vs. insulin group. HbA1c; glycated hemoglobin, GA; glycated albumin, 1,5-AG; 1,5-anhydroglucitol.

HbA1c did not significantly change in the Insulin group during the study (7.8 ± 0.7% (62 ± 8 mmol/mol) at week 0 vs. 7.8 ± 1.1% (62 ± 12 mmol/mol) at week 24, P = 0.32, Fig. 2a). In the Ins+Sita group, 17 patients (68%) underwent uptitration of sitagliptin from 50 mg to 100 mg daily at week 12. HbA1c in the Ins+Sita group was reduced from 7.9 ± 1.0% (62 ±10 mmol/mol) to 7.0 ± 0.8% (53 ± 9 mmol/mol) at week 24 (P <0.001, Fig. 2a), and a significant reduction in HbA1c was already found at week 4. There was a significant difference in the change in HbA1c at week 24 between the groups (0.1 ± 1.0% (0 ± 10 mmol/mol) in Insulin group vs. -0.9 ± 1.0% (-10 ± 11 mmol/mol) in Ins+Sita group, difference between the groups; 0.9%, 95% CI, 0.4 to 1.5%, P = 0.01). The difference in HbA1c reduction at week 24 between the groups was confirmed by MMRM (0.5%, 95% CI, 0.1 to 0.9, P = 0.01). Similarly, GA in the Ins+Sita group was significantly decreased at week 24 (23.0 ± 3.6% vs. 19.9 ± 2.4%, P <0.001), while there was no significant change in GA in the Insulin group (Fig. 2b). Serum 1,5-AG level in the Ins+Sita group, but not in the Insulin group, was significantly increased at week 24 (5.0 ± 4.1 vs. 8.6 ± 5.1 μg/mL, P <0.001, Fig. 2c). The achievement rate of HbA1c below 7.0% at week 24 was significantly higher in the Ins+Sita group than in the Insulin group (68% vs. 17%, P <0.001, Fig. 2d). Daily glycemic profile assessed by 7-point SMBG in the Insulin group was not significantly changed at week 24 compared with baseline (Fig. 2e), while postprandial glucose levels at 1 h after lunch and dinner were significantly decreased in the Ins+Sita group at week 24 (215 ± 78 vs. 179 ± 34 mg/dL after lunch, 216 ± 69 vs. 168 ± 53 mg/dL after dinner, both P <0.05, Fig. 2f).

### Body weight, hypoglycemia and other adverse events

**Fig 3:**
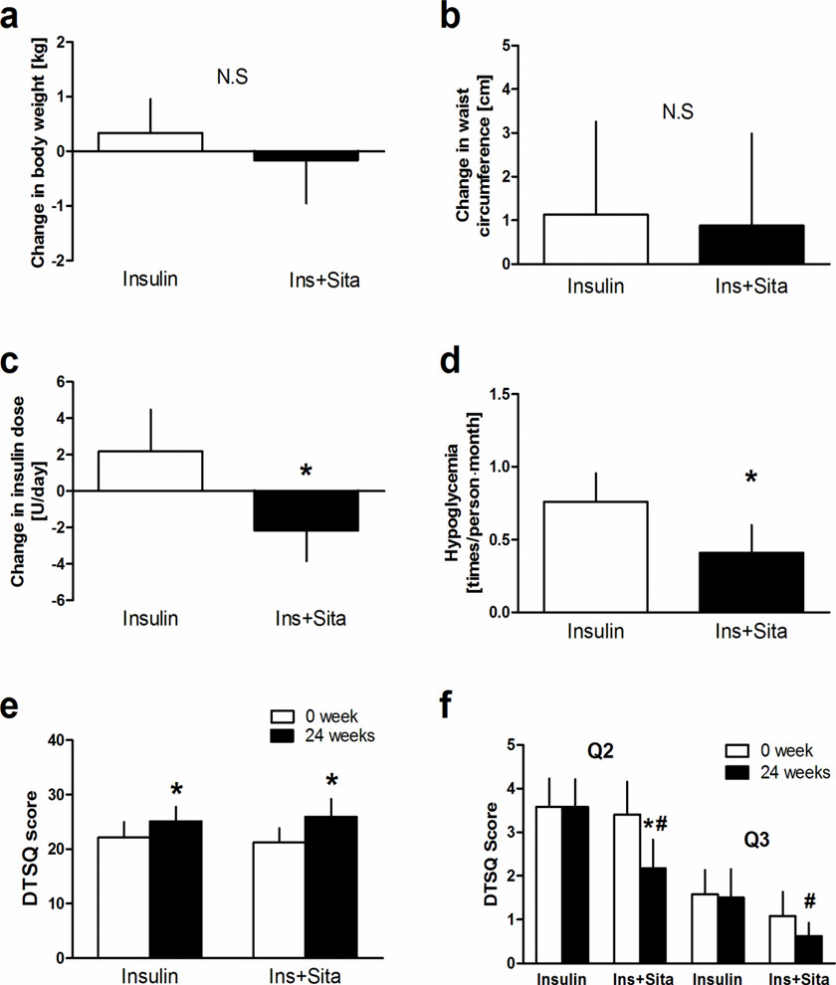
Changes in body weight (a), waist circumference (b), insulin dose (c) and incidence of hypoglycemia (d) during the study. Changes in total DTSQ score (e) and scores for question 2 (Q2; question on hyperglycemia) and question 3 (Q3; question on hypoglycemia) of DTSQ during the study (f). * *P* <0.05 and ** *P* <0.01 vs. baseline. # *P* <0.05 vs. insulin group. DTSQ; Diabetes Treatment Satisfaction Questionnaire.

There was no significant change in body weight during the study in either group (S1 Fig.), and the change in body weight at week 24 was not significantly different between the groups (0.4 ± 1.6 vs. -0.2 ± 2.0 kg, difference between the groups; -0.5 kg, 95% CI, -1.6 to 0.5, P = 0.16, Fig. 3a). There was also no significant difference in change in waist circumference at week 24 between the groups (1.13 ± 5.3 vs. 0.88 ± 5.4 cm, difference between the groups; -0.2 cm, 95% CI, -3.3 to 2.8, P = 0.94, Fig. 3b).

Daily insulin dose in the Ins+Sita group was significantly reduced compared with that in the Insulin group at week 24 (-2.2 ± 4.3 vs. 2.2 ± 8.6 U/day, difference between the groups; -4.3 U/day, 95% CI, -8.2 to -0.5, P = 0.02, Fig. 3c and S1 Fig.). The incidence of hypoglycemia in the Ins+Sita group was significantly reduced during the study compared with that in the Insulin group (0.4 ± 1.3 vs. 0.8 ± 1.3 times/person∙month, difference between the groups; -0.3 times/ person∙month, 95% CI, -0.6 to -0.1, P <0.001, Fig. 3d and S1 Fig.). There was no case of severe hypoglycemia during the study.

There was no significant change in serum alanine aminotransferase (ALT), aspartate aminotransferase (AST), gamma glutamyl transpeptidase (γ-GTP) and amylase levels in either group (data not shown). There was also no significant change in blood pressure and lipid profile during the study (data not shown).

There was no significant change in albuminuria during the study in the Ins+Sita group (median (IQR) 19.5 (1.3–105.6) vs. 45.6 (10.9–126.0) mg/g∙Cr at baseline vs. week 24, P = 0.21), whereas there was a significant increase in albuminuria in the Insulin group (median (IQR) 25.4 (6.6–74.4) vs. 38.1 (12.2–175.8) mg/g∙Cr, P = 0.002). There was no significant difference in the change in eGFR during the study between the groups (-0.3 ± 6.3 vs. -1.0 ± 6.6 mL/min/1.73 m^2^, difference between the groups; -0.7 mL/min/1.73 m^2^, 95% CI, -4.4 to 3.0, P = 0.88).

### Oxidative stress markers

There was no significant change in urinary excretion rate of 8-OHdG during the study in either group (data not shown). Urinary excretion rate of 8-iso-PGF2α in the Ins+Sita group was significantly decreased at week 24 (median (IQR) 350 (242–435) vs. 307 (225–366) pg/mg⋅Cr, P = 0.01), whereas it was not significantly changed at week 24 in the Insulin group (median (IQR) 311 (190–347) vs. 329 (240–507) pg/mg⋅Cr, P = 0.27).

### Treatment satisfaction

Total DTSQ score was significantly increased at week 24 in both groups (22.2 ± 7.1 vs. 25.1 ± 6.9, median (IQR) 20.5 (17.0–29.0) vs. 25.5 (18.0–30.0) in Insulin group and 21.2 ± 6.7 vs. 25.9 ± 8.5, median (IQR) 21.0 (16.5–24.0) vs. 27.0 (21.5–32.5) in Ins+Sita group, both P <0.05, Fig. 3e). Question 2 of DTSQ (question on hyperglycemia) in the Ins+Sita group showed a significant improvement at week 24 (3.4 ± 2.0 vs. 2.2 ± 1.6, median (IQR) 3.0 (2.0–5.0) vs. 2.0 (1.0–3.75), P = 0.02), and there was a significant difference between the groups at week 24 (2.2 ± 1.6 vs. 3.6 ± 1.7, median (IQR) 2.0 (1.0–3.75) vs. 3.5 (3.0–5.00), P = 0.01, Fig. 3f). Question 3 of DTSQ (question on hypoglycemia) showed a significant improvement at week 24 in the Ins+Sita group compared with the Insulin group (1.1 ± 1.4 vs. 0.6 ± 0.8, median (IQR) 0.0 (0.0–2.0) vs. 0.0 (0.0–1.0), P = 0.04, Fig. 3f). There was no significant change in DiabMedSat score during the study in either group (data not shown).

### Meal tolerance test (MTT)

**Fig 4:**
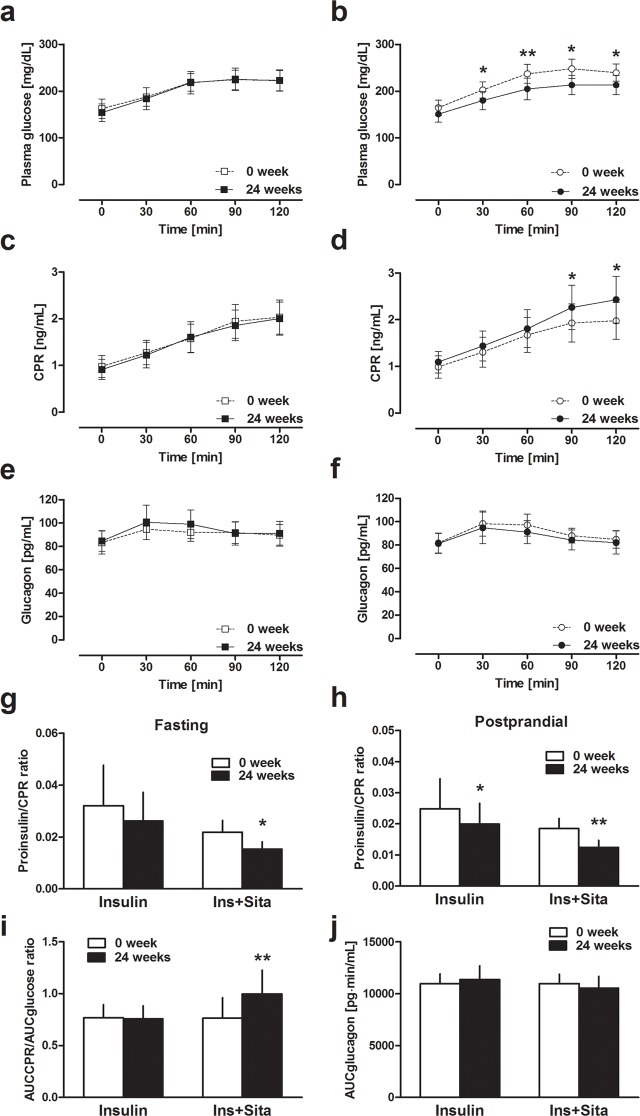
Changes in plasma glucose, CPR, and glucagon during MTT between 0 and 24 weeks in insulin group (a, c, e) and insulin plus sitagliptin group (b, d, f). Changes in fasting (g) and postprandial (h) proinsulin to CPR ratio during MTT between 0 and 24 weeks. Changes in AUCCPR/AUCglucose (i) and AUCglucagon (j) during MTT between 0 and 24 weeks. * *P* <0.05 and ** *P* <0.01 vs. baseline. CPR: C-peptide immunoreactivity, MTT; meal tolerance test, AUC: area under the curve.

Plasma glucose, CPR, and glucagon levels during MTT at weeks 0 and 24 are shown in Fig. 4. There was no significant change in plasma glucose and CPR levels in the Insulin group at week 24. In the Ins+Sita group, plasma glucose levels at 30, 60, 90 and 120 min after meal ingestion were significantly decreased at week 24 (all P <0.05). CPR levels at 90 and 120 min were significantly increased at week 24 in the Ins+Sita group (median (IQR) 1.70 (1.35–2.55) vs. 2.30 (1.50–2.80) ng/mL, P = 0.02 and 1.80 (1.55–2.45) vs. 2.60 (1.55–3.20) ng/mL, P = 0.03, respectively). There was no significant change in plasma glucagon level during MTT at week 24 in either group.

Fasting and 60 min postprandial proinsulin/CPR ratio in the Ins+Sita group were both significantly decreased at week 24 (both P <0.05, Figs. 4g and 4h), while only postprandial proinsulin/CPR ratio was significantly decreased in the Insulin group. AUCCPR/AUCglucose ratio in the Ins+Sita group was also significantly increased at week 24 (median (IQR) 0.68 (0.48–0.96) vs. 0.83 (0.74–1.25), P <0.001, Fig. 4i), although there was no change in the Insulin group. Although AUCglucagon in the Ins+Sita group tended to be decreased at week 24, the difference was not significant (median (IQR) 10875 (8797–12960) vs. 10290 (8295–12645) pg∙min/mL, P = 0.31, Fig. 4j). Intact and total GLP-1 levels at 0 and 60 min were significantly increased at week 24 in the Ins+Sita group (S2 Fig.), whereas there was no significant change in total GIP level at week 24 (S2 Fig.).

### Predictors of change in HbA1c after sitagliptin add-on treatment

Correlations between change in HbA1c during the study (ΔHbA1c) and other parameters are shown in Table 2. There were significant correlations between ΔHbA1c and baseline HbA1c (r = -0.49, P = 0.01, S3 Fig.), GA (r = -0.60, P = 0.01, S3 Fig.) and 1,5-AG (r = 0.57, P = 0.01, S3 Fig.) in the Ins+Sita group. Baseline fasting CPR (r = -0.51, P = 0.01, S3 Fig.), postprandial CPR (r = -0.50, P = 0.01), Δ_0–120min_CPR (r = -0.45, P = 0.02) and fasting intact proinsulin (r = -0.56, P = 0.01) were also significantly correlated with ΔHbA1c in the Ins+Sita group. Also, change in AUCCPR/AUCglucose ratio during the study (ΔAUCCPR/AUCglucose ratio) was significantly correlated with ΔHbA1c in the Ins+Sita group (r = -0.49, P = 0.01). A significant correlation between ΔHbA1c and baseline GA or 1,5-AG was also observed in the Insulin group (S3 Fig.) as well as in total subjects (Table 2).

**Table 2:** Correlation between change in HbA1c during the study (ΔHbA1c) and clinical parameters.

	Insulin		Ins+ Sita		Total	
	R	*P*	R	*P*	R	*P*
Baseline parameters						
Age (years)	-0.17	0.43	-0.27	0.19	-0.19	0.18
Duration of diabetes (years)	-0.30	0.15	0.22	0.29	-0.04	0.78
BMI (kg/m^2^)	0.27	0.20	0.05	0.81	0.20	0.18
Duration of insulin therapy (years)	-0.17	0.43	0.25	0.23	-0.04	0.81
Insulin dose (U/kg/day)	0.01	0.96	0.37	0.07	0.18	0.22
HbA1c (%)	-0.09	0.68	-0.49	0.01	-0.24	0.10
GA (%)	-0.55	<0.01	-0.60	<0.01	-0.51	<0.01
1,5-AG (%)	0.49	0.02	0.57	<0.01	0.55	<0.01
Fasting CPR (ng/mL)	0.08	0.72	-0.51	<0.01	-0.26	0.07
120 min postprandial CPR (ng/mL)	0.22	0.30	-0.50	0.01	-0.17	0.25
Fasting CPR index	0.36	0.09	-0.33	0.11	-0.04	0.98
AUCCPR/AUCglucose ratio	0.39	0.66	-0.36	0.07	-0.03	0.86
Δ_0–120min_CPR	0.34	0.10	-0.45	0.02	-0.13	0.36
Fasting glucagon (pg/mL)	0.05	0.83	0.08	0.71	0.05	0.73
AUCglucagon(pg∙min/mL)	-0.57	0.79	-0.30	0.89	-0.35	0.81
Intact proinsulin	0.10	0.64	-0.56	<0.01	-0.22	0.12
Intact proinsulin/CPR ratio	0.16	0.46	-0.33	0.11	-0.11	0.46
Δ_0–120min_Intact GLP-1	-0.21	0.33	0.13	0.52	-0.07	0.62
Change in parameters during the study						
ΔFasting CPR	0.16	0.45	0.17	0.43	0.09	0.54
Δ120min postprandial CPR	0.11	0.60	0.26	0.90	-0.05	0.73
ΔAUCCPR/AUCglucose ratio	0.25	0.24	-0.49	0.01	-0.30	0.04
ΔΔ_0–120min_CPR	0.37	0.08	-0.30	0.15	-0.09	0.52
ΔFasting glucagon	-0.02	0.93	-0.11	0.60	-0.05	0.75
ΔAUCglucagon (pg∙min/mL)	0.13	0.54	0.45	0.83	0.11	0.45

BMI; body mass index, HbA1c; glycated hemoglobin, GA; glycated albumin, 1,5-AG; 1,5-anhydroglucitol, CPR; C-peptide immunoreactivity, AUC; area under the curve.

Multivariate regression analysis including age, sex, duration of diabetes, BMI, insulin dose, baseline HbA1c and baseline fasting CPR showed insulin dose, baseline HbA1c and baseline fasting CPR to be independent variables predicting ΔHbA1c in the Ins+Sita group (standardized coefficients; 0.54, -0.57 and -0.44, P = 0.01, 0.002 and 0.04, respectively).

## Discussion

In this study, adding sitagliptin effectively reduced HbA1c by 0.9% without an increased risk of hypoglycemia or weight gain in Japanese patients with T2DM whose glycemic control was suboptimal despite at least twice daily injections of insulin. On the other hand, there was no significant improvement in HbA1c in the Insulin group, in which physicians tried to achieve the glycemic goal by adjustment of insulin dose. Improvement of glycemic control by adding sitagliptin was also confirmed by a significant reduction in GA and a concomitant increase in 1,5-AG during the study. The reduction in HbA1c in this study was comparable or greater than that in previous studies conducted in USA or Europe (0.5–0.7%) despite the comparable baseline HbA1c level[9–11], which was consistent with the previous report showing a greater glucose-lowering effect of DPP-4 inhibitors in Asians compared with Caucasians[12]. Moreover, in this study, we also examined the daily glycemic profile by assessing 7-point SMBG, and showed that adding sitagliptin to insulin significantly reduced the postprandial glucose level.

Vilsboll et al. have reported that adding sitagliptin to insulin increased the incidence of hypoglycemia in patients with T2DM [9]. The improvement of glycemic control by adding sitagliptin without an increased incidence of hypoglycemia observed in this study might be due to difference in the study protocol. In this study, insulin dose was reduced if initial HbA1c level was less than 8%, and the concomitant use of insulin secretagogues was discontinued during the study. Although the attending physician was able to adjust insulin dose during the study, the daily insulin dose remained significantly lower in the Ins+Sita group compared with the Insulin group. Thus, our results suggest that reducing the insulin dose by up to 20% and discontinuing insulin secretagogues may prevent hypoglycemia when a DPP-4 inhibitor is added to insulin therapy. Hong et al. have also recently reported the efficacy and safety of adding sitagliptin in patients with T2DM whose HbA1c was poorly controlled with insulin[21]. Adding sitagliptin resulted in a 0.6% reduction in HbA1c without an increase in incidence of hypoglycemia compared with the control group in which insulin dose was up-titrated by 25% during the study.

Treatment satisfaction assessed by DTSQ was also improved in the Ins+Sita group, which was mainly derived from the improvement of patients’ satisfaction with controlling hyper- and hypoglycemia. On the other hand, there was no change in DiabMedSat. The reason for this inconsistency is unclear, but the results of the latter might be affected by ceiling effects[22]. Since there was no serious adverse event during the study, consistent with prior reports[23–25], our results proved that adding sitagliptin to insulin effectively and safely improves glycemic control and patients’ satisfaction in Japanese patients with T2DM. In addition, in this study, sitagliptin treatment showed some reduction in oxidative stress markers, which may also be favorable to prevent the development or progression of vascular complications, although a favorable effect of DPP-4 inhibitors on cardiovascular outcome remains to be established[26, 27].

To explore the mechanism by which sitagliptin improved glycemic control, MTT was also performed. As a result, MTT revealed that adding sitagliptin decreased postprandial glycemic excursion through enhancing postprandial insulin secretion. This change was accompanied by an increase in intact GLP-1 level after adding sitagliptin, confirming enhancement of incretin action. On the other hand, we did not observe a significant change in glucagon level after adding sitagliptin. It has been reported that DPP-4 inhibitors suppress glucagon level[28–30]. This inconsistency may be in part due to the inaccuracy of current methods of glucagon measurement[31]. It has been reported that GLP-1 suppresses glucagon secretion through enhancing insulin secretion[32]. Thus, the insufficient glucagon suppression observed in this study may have been due to marked beta cell dysfunction in our patients with diabetes duration of about 20 years, although it has also been reported that GLP-1 suppresses glucagon secretion through stimulating somatostatin secretion from delta cells[33].

Finally, we tried to explore the predictors of HbA1c reduction by adding sitagliptin. As a result, baseline HbA1c level was a strong predictor of HbA1c reduction, consistent with previous reports[34, 35]. However, baseline GA and 1,5-AG levels were more strongly correlated with HbA1c reduction by adding sitagliptin than with baseline HbA1c, and were also associated with HbA1c reduction in the Insulin group as well as in all patients, suggesting that baseline GA and 1,5-AG levels similarly or even more sensitively predict a reduction in HbA1c after intensification of treatment than does baseline HbA1c. Our study also revealed that baseline beta cell function and improvement of beta cell function during the study were also predictors of HbA1c reduction after adding sitagliptin. It has been reported that baseline beta cell function and its progressive decline are important predictors of future glycemic control in patients with T2DM[36–39]. Thus, these findings suggest that beta cell function as well as baseline HbA1c level is a major contributor to the glucose-lowering effect of sitagliptin added to insulin in Japanese patients with T2DM.

The small sample size and open-labeled design are limitations of this study. However, this study was conducted at a single institution and all attending physicians were specialists in diabetes, reflecting the “real world” clinical practice setting. Treatment allocation was blinded to both the physicians and patients until day 0 to avoid selection bias, and both groups similarly received standard care during the study. In addition, half of the patients were also randomized to a control (Insulin) group; therefore, we believe that we could at least partly eliminate the effects of study enrollment by comparison to the control group. In this study, we did not use a strict algorithm for insulin titration during the study and the insulin dose was mainly adjusted by the attending physicians at each visit, also reflecting the “real world” usual care setting. This may be the reason that the change in insulin dose in this study was relatively small. Recent studies have suggested that self-titration of insulin dose by patients based on their SMBG values resulted in better glycemic control compared with titration by physicians[40, 41]. Thus, introducing self-titration of insulin dose by patients might have resulted in better glycemic control in this study, although this would have been the case in both groups. On the other hand, comprehensive assessments including MTT and treatment satisfaction in addition to other clinical outcomes were a strength of this study, which allowed us to better understand the efficacy and safety of sitagliptin added to insulin therapy.

In conclusion, adding sitagliptin to insulin reduced HbA1c level by 0.9% without weight gain or increase in hypoglycemia, and improved treatment satisfaction in Japanese patients with suboptimally controlled T2DM. Adding a DPP-4 inhibitor may be an option when the glycemic goal is not achieved by at least twice daily injection of insulin. Longer-term efficacy and safety should be tested in future studies.

## Supporting Information

S1 CONSORT Checklist (DOC)

S1 Fig Changes in body weight (a), insulin dose (b) and incidence of hypoglycemia (c) at each month during the study. * *P* <0.05 vs. insulin group.
(TIF)

S2 Fig Changes in triglycerides, free fatty acids, intact GLP-1, total GLP-1 and total GIP during MTT between 0 and 24 weeks in insulin group (a, c, e, g, i) and insulin plus sitagliptin group (b, d, f, h, j). * *P* <0.05 vs. baseline. MTT; meal tolerance test.
(TIF)

S3 Fig Correlations between change in HbA1c during the study and baseline HbA1c (a), GA (b), 1,5-AG (c) and fasting CPR (d). Open circles and solid lines; insulin group, closed circles and dotted lines; insulin plus sitagliptin group. HbA1c; glycated hemoglobin, GA; glycated albumin, 1,5-AG; 1,5-anhydroglucitol, CPR: C-peptide immunoreactivity.
(TIF)

S1 IRB permission (PDF)

S1 Protocol Original version. (DOCX)

S2 Protocol English version. (DOCX)

## References

[1] Kahn SE, Cooper ME, Del Prato S. Pathophysiology and treatment of type 2 diabetes: perspectives on the past, present, and future. Lancet. 2014; 383: 1068–1083. 10.1016/S0140-6736(13)62154-6 24315620 PMC4226760

[2] Home P, Riddle M, Cefalu WT, Bailey CJ, Bretzel RG, del Prato S, et al Insulin Therapy in People With Type 2 Diabetes: Opportunities and Challenges? Diabetes Care. 2014; 37: 1499–1508. 10.2337/dc13-2743 24855154 PMC5131884

[3] Home PD, Shen C, Hasan MI, Latif ZA, Chen JW, Gonzalez Galvez G. Predictive and Explanatory Factors of Change in HbA1c in a 24-Week Observational Study of 66,726 People With Type 2 Diabetes Starting Insulin Analogs. Diabetes Care. 2014; 37: 1237–1245. 10.2337/dc13-2413 24595628

[4] Giugliano D, Maiorino MI, Bellastella G, Chiodini P, Ceriello A, Esposito K. Efficacy of Insulin Analogs in Achieving the Hemoglobin A1c Target of <7% in Type 2 Diabetes: Meta-analysis of randomized controlled trials. Diabetes Care. 2011; 34: 510–517. 10.2337/dc10-1710 21216850 PMC3024378

[5] Drucker DJ, Nauck MA. The incretin system: glucagon-like peptide-1 receptor agonists and dipeptidyl peptidase-4 inhibitors in type 2 diabetes. Lancet. 2006; 368: 1696–1705. 17098089 10.1016/S0140-6736(06)69705-5

[6] Karagiannis T, Paschos P, Paletas K, Matthews DR, Tsapas A. Dipeptidyl peptidase-4 inhibitors for treatment of type 2 diabetes mellitus in the clinical setting: systematic review and meta-analysis. BMJ. 2012; 344: e1369 10.1136/bmj.e1369 22411919

[7] Farngren J, Persson M, Schweizer A, Foley JE, Ahren B. Glucagon dynamics during hypoglycaemia and food-re-challenge following treatment with vildagliptin in insulin-treated patients with type 2 diabetes. Diabetes Obes Metab. 2014; 16: 812–818. 10.1111/dom.12284 24612221

[8] Vilsboll T. The effects of glucagon-like peptide-1 on the beta cell. Diabetes Obes Metab. 2009; 11 Suppl 3: 11–18. 10.1111/j.1463-1326.2009.01073.x 19878257

[9] Vilsboll T, Rosenstock J, Yki-Jarvinen H, Cefalu WT, Chen Y, Luo E, et al Efficacy and safety of sitagliptin when added to insulin therapy in patients with type 2 diabetes. Diabetes Obes Metab. 2010; 12: 167–177. 10.1111/j.1463-1326.2009.01173.x 20092585

[10] Fonseca V, Schweizer A, Albrecht D, Baron MA, Chang I, Dejager S. Addition of vildagliptin to insulin improves glycaemic control in type 2 diabetes. Diabetologia. 2007; 50: 1148–1155. 17387446 10.1007/s00125-007-0633-0

[11] Rosenstock J, Rendell MS, Gross JL, Fleck PR, Wilson CA, Mekki Q. Alogliptin added to insulin therapy in patients with type 2 diabetes reduces HbA(1C) without causing weight gain or increased hypoglycaemia. Diabetes Obes Metab. 2009; 11: 1145–1152. 10.1111/j.1463-1326.2009.01124.x 19758359

[12] Kim YG, Hahn S, Oh TJ, Kwak SH, Park KS, Cho YM. Differences in the glucose-lowering efficacy of dipeptidyl peptidase-4 inhibitors between Asians and non-Asians: a systematic review and meta-analysis. Diabetologia. 2013; 56: 696–708. 10.1007/s00125-012-2827-3 23344728

[13] Japan Diabetes Society. The guideline for the treatment of diabetes. Tokyo: Japan Diabetes Society; 2014.

[14] Ashwell SG, Bradley C, Stephens JW, Witthaus E, Home PD. Treatment satisfaction and quality of life with insulin glargine plus insulin lispro compared with NPH insulin plus unmodified human insulin in individuals with type 1 diabetes. Diabetes Care. 2008; 31: 1112–1117. 10.2337/dc07-1183 18339977

[15] Ishii H, Iwase M, Seino H, Shuto Y, Atsumi Y. Assessment of quality of life in patients with type 2 diabetes mellitus before and after starting biphasic insulin aspart 30 (BIAsp 30) therapy: IMPROVE study in Japan. Curr Med Res Opin. 2011; 27: 643–650. 10.1185/03007995.2010.551760 21250861

[16] Saisho Y, Tanaka K, Abe T, Kawai T, Itoh H. Lower beta cell function relates to sustained higher glycated albumin to glycated hemoglobin ratio in Japanese patients with type 2 diabetes. Endocr J. 2014; 61: 149–157. 24212881 10.1507/endocrj.ej13-0376

[17] Kodani N, Saisho Y, Tanaka K, Kawai T, Itoh H. Effects of mitiglinide, a short-acting insulin secretagogue, on daily glycemic variability and oxidative stress markers in Japanese patients with type 2 diabetes mellitus. Clin Drug Investig. 2013; 33: 563–570. 23797928 10.1007/s40261-013-0098-5

[18] Committee on the Standardization of Diabetes Mellitus-Related Laboratory Testing of Japan Diabetes Society. International clinical harmonization of glycated hemoglobin in Japan: From Japan Diabetes Society to National Glycohemoglobin Standardization Program values. Diabetol Int. 2012; 3: 8–10. 10.1111/j.2040-1124.2012.00207.x PMC4014931 24843544

[19] Yabe D, Watanabe K, Sugawara K, Kuwata H, Kitamoto Y, Sugizaki K, et al Comparison of incretin immunoassays with or without plasma extraction: Incretin secretion in Japanese patients with type 2 diabetes. Journal of diabetes investigation. 2012; 3: 70–79. 10.1111/j.2040-1124.2011.00141.x 24843548 PMC4014935

[20] Matsuo S, Imai E, Horio M, Yasuda Y, Tomita K, Nitta K, et al Revised equations for estimated GFR from serum creatinine in Japan. Am J Kidney Dis. 2009; 53: 982–992. 10.1053/j.ajkd.2008.12.034 19339088

[21] Hong ES, Khang AR, Yoon JW, Kang SM, Choi SH, Park KS, et al Comparison between sitagliptin as add-on therapy to insulin and insulin dose-increase therapy in uncontrolled Korean type 2 diabetes: CSI study. Diabetes Obes Metab. 2012; 14: 795–802. 10.1111/j.1463-1326.2012.01600.x 22443183

[22] Bradley C. Diabetes treatment satisfaction questionnaire. Change version for use alongside status version provides appropriate solution where ceiling effects occur. Diabetes Care. 1999; 22: 530–532. 10097946 10.2337/diacare.22.3.530

[23] Goossen K, Graber S. Longer term safety of dipeptidyl peptidase-4 inhibitors in patients with type 2 diabetes mellitus: systematic review and meta-analysis. Diabetes Obes Metab. 2012; 14: 1061–1072. 10.1111/j.1463-1326.2012.01610.x 22519906

[24] Eurich DT, Simpson S, Senthilselvan A, Asche CV, Sandhu-Minhas JK, McAlister FA. Comparative safety and effectiveness of sitagliptin in patients with type 2 diabetes: retrospective population based cohort study. BMJ. 2013; 346: f2267 10.1136/bmj.f2267 23618722 PMC3635468

[25] Monami M, Dicembrini I, Martelli D, Mannucci E. Safety of dipeptidyl peptidase-4 inhibitors: a meta-analysis of randomized clinical trials. Curr Med Res Opin. 2011; 27 Suppl 3: 57–64. 10.1185/03007995.2011.602964 22106978

[26] White WB, Cannon CP, Heller SR, Nissen SE, Bergenstal RM, Bakris GL, et al Alogliptin after acute coronary syndrome in patients with type 2 diabetes. N Engl J Med. 2013; 369: 1327–1335. 10.1056/NEJMoa1305889 23992602

[27] Scirica BM, Bhatt DL, Braunwald E, Steg PG, Davidson J, Hirshberg B, et al Saxagliptin and cardiovascular outcomes in patients with type 2 diabetes mellitus. N Engl J Med. 2013; 369: 1317–1326. 10.1056/NEJMoa1307684 23992601

[28] Herman GA, Bergman A, Stevens C, Kotey P, Yi B, Zhao P, et al Effect of single oral doses of sitagliptin, a dipeptidyl peptidase-4 inhibitor, on incretin and plasma glucose levels after an oral glucose tolerance test in patients with type 2 diabetes. J Clin Endocrinol Metab. 2006; 91: 4612–4619. 16912128 10.1210/jc.2006-1009

[29] Muscelli E, Casolaro A, Gastaldelli A, Mari A, Seghieri G, Astiarraga B, et al Mechanisms for the antihyperglycemic effect of sitagliptin in patients with type 2 diabetes. J Clin Endocrinol Metab. 2012; 97: 2818–2826. 10.1210/jc.2012-1205 22685234

[30] Solis-Herrera C, Triplitt C, Garduno-Garcia Jde J, Adams J, DeFronzo RA, Cersosimo E. Mechanisms of glucose lowering of dipeptidyl peptidase-4 inhibitor sitagliptin when used alone or with metformin in type 2 diabetes: a double-tracer study. Diabetes Care. 2013; 36: 2756–2762. 10.2337/dc12-2072 23579178 PMC3747902

[31] Bak MJ, Albrechtsen NW, Pedersen J, Hartmann B, Christensen M, Vilsboll T, et al Specificity and sensitivity of commercially available assays for glucagon and oxyntomodulin measurement in humans. Eur J Endocrinol. 2014; 170: 529–538. 10.1530/EJE-13-0941 24412928

[32] Cooperberg BA, Cryer PE. Beta-cell-mediated signaling predominates over direct alpha-cell signaling in the regulation of glucagon secretion in humans. Diabetes Care. 2009; 32: 2275–2280. 10.2337/dc09-0798 19729529 PMC2782990

[33] de Heer J, Rasmussen C, Coy DH, Holst JJ. Glucagon-like peptide-1, but not glucose-dependent insulinotropic peptide, inhibits glucagon secretion via somatostatin (receptor subtype 2) in the perfused rat pancreas. Diabetologia. 2008; 51: 2263–2270. 10.1007/s00125-008-1149-y 18795252

[34] DeFronzo RA, Stonehouse AH, Han J, Wintle ME. Relationship of baseline HbA1c and efficacy of current glucose-lowering therapies: a meta-analysis of randomized clinical trials. Diabet Med. 2010; 27: 309–317. 10.1111/j.1464-5491.2010.02941.x 20536494

[35] Esposito K, Chiodini P, Bellastella G, Maiorino MI, Giugliano D. Proportion of patients at HbA1c target <7% with eight classes of antidiabetic drugs in type 2 diabetes: systematic review of 218 randomized controlled trials with 78 945 patients. Diabetes Obes Metab. 2012; 14: 228–233. 10.1111/j.1463-1326.2011.01512.x 21958121

[36] Matthews DR, Cull CA, Stratton IM, Holman RR, Turner RC. UKPDS 26: Sulphonylurea failure in non-insulin-dependent diabetic patients over six years. UK Prospective Diabetes Study (UKPDS) Group. Diabet Med. 1998; 15: 297–303. 9585394 10.1002/(SICI)1096-9136(199804)15:4<297::AID-DIA572>3.0.CO;2-W

[37] Kahn SE, Lachin JM, Zinman B, Haffner SM, Aftring RP, Paul G, et al Effects of rosiglitazone, glyburide, and metformin on beta-cell function and insulin sensitivity in ADOPT. Diabetes. 2011; 60: 1552–1560. 10.2337/db10-1392 21415383 PMC3292330

[38] TODAY Study Group. Effects of Metformin, Metformin Plus Rosiglitazone, and Metformin Plus Lifestyle on Insulin Sensitivity and beta-Cell Function in TODAY. Diabetes Care. 2013; 36: 1749–1757. 10.2337/dc12-2393 23704674 PMC3661836

[39] Saisho Y, Kou K, Tanaka K, Abe T, Shimada A, Kawai T, et al Association between beta cell function and future glycemic control in patients with type 2 diabetes. Endocr J. 2013; 60: 517–523. 23268927

[40] Davies M, Storms F, Shutler S, Bianchi-Biscay M, Gomis R. Improvement of glycemic control in subjects with poorly controlled type 2 diabetes: comparison of two treatment algorithms using insulin glargine. Diabetes Care. 2005; 28: 1282–1288. 15920040 10.2337/diacare.28.6.1282

[41] Harris SB, Yale JF, Berard L, Stewart J, Abbaszadeh B, Webster-Bogaert S, et al Does a patient-managed insulin intensification strategy with insulin glargine and insulin glulisine provide similar glycemic control as a physician-managed strategy? Results of the START (Self-Titration With Apidra to Reach Target) Study: a randomized noninferiority trial. Diabetes Care. 2014; 37: 604–610. 10.2337/dc13-1636 24170757

