# Supplementary material for: Efficacy and Safety of Sitagliptin Added to Insulin in Japanese Patients with Type 2 Diabetes: The EDIT Randomized Trial
Source: PLoS One. 2015 Mar 27;10(3):e0121988. doi: 10.1371/journal.pone.0121988 (PMC4376939; doi:10.1371/journal.pone.0121988)
Supplement: S1 IRB permission — (PDF) [file pone.0121988.s005.pdf]

倫理審査結果通知書

2013年3月1日

内科学(腎臓・内分泌・代謝)
伊藤 裕 様

慶應義塾大学医学部長
末松 誠

過日ご提出されました倫理審査申請についての、倫理委員会による審査の結果、下記のとおり判定されましたので、お知らせいたします。

記

受付番号 : 2010-175-3

課題名 : 2型糖尿病患者におけるDPP-4阻害薬とインスリンとの併用効果についての検討

承認(許可)日 : 2013年2月25日

承認番号 : 20100175

研究期間 : 2010 年 12 月 01 日 ～ 2016 年 03 月 31 日
※承認番号、研究期間は、判定が「承認」の場合のみ有効

判定 : 承認

勧告または理由 : 審査の結果、承認いたしました。

＊ 審査結果が「承認」の場合は、裏面の注意書きをお読みください。 以上
