# Supplementary material for: Efficacy and Safety of Sitagliptin Added to Insulin in Japanese Patients with Type 2 Diabetes: The EDIT Randomized Trial
Source: PLoS One. 2015 Mar 27;10(3):e0121988. doi: 10.1371/journal.pone.0121988 (PMC4376939; doi:10.1371/journal.pone.0121988)
Supplement: S1 Protocol — (DOCX) [file pone.0121988.s006.docx]

研究計画の詳細（疫学手法を用いて解析する研究の場合）

1　研究目的

2型糖尿病の最適な治療を目指して、１日２回注射以上の強化インスリン療法施行中にも関わらず血糖コントロールが不十分な２型糖尿病患者へのDPP-4阻害薬シタグリプチンの併用効果を検討する。

2 研究デザインのタイプ

前向き非盲検無作為群間比較法

（prospective, open, randomized and comparative trial）

試験のアウトライン（研究期間 ： 6ヶ月）

食事負荷試験

^※1^ HbA1cが7.5％未満の場合はインスリン投与量を20％減量する。

^※2^ シタグリプチンは50 mgから実施し、3ヶ月後に効果不十分な場合は、100 mgに増量する。

Lab　：　血液生化学検査、尿検査

食事負荷試験 ： テストミール負荷試験

A群　：　強化インスリン療法^※1^＋シタグリプチン併用群（25例）^※2^

0M 2W 1M 2M 3M 4M 5M 6M

B群　：　強化インスリン療法継続群（25例）

同意・割付

Lab

Lab

Lab

Lab

観察期

-1M

（強化インスリン療法）

食事負荷試験

Lab

Lab

Lab

研究薬の投与方法

1）観察期間（1ケ月以上）

研究担当医師は過去の診療記録から、１日２回注射以上の強化インスリン療法を行なっている血糖コントロール不十分（HbA1c 6.5％以上）の対象患者を選定し、試験内容に関し倫理委員会で承認された説明文書を用いて説明し、文書による同意を得て、A群：シタグリプチン併用群とB群：強化インスリン療法継続群に無作為割付を行う。

2）研究期間（6ヶ月）

研究担当医師は割付の結果に従い、下記の治療を開始する。

A群：シタグリプチン併用群

シタグリプチン 50 mg/日を追加投与するが、その際に、HbA1cが7.5％未満の場合は、インスリン投与量を20％減量する。その後インスリン量は日本糖尿病学会の指針に従い、空腹時血糖１３０mg/dl未満、食後２時間血糖１８０mg/ｄｌ未満を目標に責任インスリンのアルゴリズムに基づき主治医の指示で適宜調節を行う。試験開始より3ヶ月以降で、目標血糖コントロールレベルであるHbA1c 6.5％に未達の場合は、低血糖を含む忍容性に注意した上で、シタグリプチンを100 mgまで増量する。

B群：強化インスリン療法群

強化インスリン療法を継続する。インスリン量は日本糖尿病学会の指針に従い、空腹時血糖１３０mg/dl未満、食後２時間血糖１８０mg/ｄｌ未満を目標に責任インスリンのアルゴリズムに基づき主治医の指示で適宜調節を行う。

^注）^  低血糖が起こった場合は、A群、B群ともに、インスリンの減量や投与回数を減らす等の適切な処置を行なう。

併用薬に関する規定

他の経口糖尿病治療薬を使用中の患者については、シタグリプチン併用群に割り当てられた患者では、低血糖の可能性を考慮し、インスリン分泌促進薬（スルフォニル尿素薬、グリニド薬）の投与は試験開始時より中止とする。その他の場合は試験中継続可とするが、試験中は可能な限り変更は行わない。

合併症治療薬（抗血小板薬、高血圧治療薬、高脂血症治療薬等）は制限しないが介入期間中は、可能な限り薬剤の用量変更、新規追加は行わない。

症例登録・割付方法

登録目標症例数は、各群25例とし、観察期間終了後に下記の2群に無作為に割り付ける。割り付けは、登録時の患者背景因子（HbA1c値、性別、年齢、BMI、インスリン投与回数^※^）を割付因子として、コンピュータを用いた無作為割り付け（最小化法）を実施する。

A群 ： 強化インスリン療法 ＋ シタグリプチン併用群

B群　： 強化インスリン療法継続群

^※^ 頻回注射（インスリン注射4回以上）とその他（インスリン注射3回以下）に分ける。

3　結果（アウトカム）と原因（曝露）に関する指標

3.1 結果（アウトカム）の指標

主要評価項目

治療6ヶ月後におけるHbA1c値の治療開始時からの変化量・変化率

副次評価項目

- 1. 食事負荷（テストミール負荷）時における下記項目の推移および曲線下面積（AUC）、治療6ヶ月後における各項目AUCの治療開始時からの変化量・変化率
     - 血糖
     - C-ペプチド（CPR）
     - インタクトプロインスリン
     - グルカゴン
     - 遊離脂肪酸(free fatty acid, FFA)
- Active GLP-1
- Total GIP
- トリグリセライド
  1. 治療6ヶ月後の治療開始時からの下記項目の変化量・変化率
- 腹囲
- 自己血糖測定（SMBG）による一日血糖プロファイル
- QOLアンケート調査（糖尿病治療満足度調査：DTSQおよびDiabMedSat）
- 酸化ストレスマーカー（尿中8-イソプロスタン、尿中8-OHdG）
  1. 治療1、2、3、4、5、6ヶ月後の治療開始時からの下記項目の変化量・変化率
- 血圧
- 体重
- HbA1c
- グリコアルブミン
- １,５-AG
- C-ペプチド（CPR）
- インスリン投与量、投与回数
- 問診表およびSMBGによる低血糖の頻度
  1. サブ解析項目（探索的解析）

治療期間終了後、年齢、性別、HbA1c、BMI、インスリン投与法などの層別解析（群内変動、群間比較）を検討し、探索的解析を行う。

- 1. 安全性評価

副作用発現率（全ての有害事象の発現と試験薬と関連性が否定できない臨床検査異常や副作用の発現）

4 研究対象者

4.1 研究対象者となる可能性のある集団の全体

慶應義塾大学病院内科に外来通院中の2型糖尿病患者

4.2 取込（採用）基準 （比較群についても記載）

慶應義塾大学病院に通院中の、1日2回注射以上の強化インスリン療法を２カ月以上行なっているにも関わらず血糖コントロールが不十分（HbA1c値が6.5％以上）の2型糖尿病患者で、下記に該当する患者

選択基準

以下の基準を全て満たす患者を対象とする。

- 過去2ヶ月において、経口糖尿病治療薬を変更していない患者
- 20歳以上の患者
- 性別不問
- 外来患者のみ
- 文書による同意を得られた患者

4.3 除外基準 （比較群についても記載）

以下のいずれかに抵触する患者は本研究に組み入れないこととする。

- 1型糖尿病患者
- 中等度以上の腎機能障害の患者

（男性でsCr 1.5 mg/dL以上、女性 sCr 1.3 mg/dL以上またはeGFR 30ｍL/min未満を目安とする）

- 重篤な肝障害の患者

（AST、ALTの基準値上限の３倍以上を目安とする）

- その他研究担当医師が不適格と判断した患者

4.4 サンプル数およびその算出根拠

介入群　：　強化インスリン療法＋シタグリプチン50mg1日1回併用　25症例

非介入群　：　強化インスリン療法　25症例

計50例（各群25例）

海外の臨床成績^1)^ より、シタグリプチン＋インスリン併用療法群およびインスリン療法群におけるHbA1c減少量を、それぞれ-0.7±0.7％、-0.1±0.7％と推定すると、危険率α＝0.05、件出力1－β＝0.80の統計学的条件下で両群間における有意差を検出するための必要最小症例数は、各群23例、合計46例となる。脱落症例等を考慮し、各群25例、合計50例を登録目標症例数と設定する。

1. T Vilsvoll et al. *Diabetecs Obesity and Metabolism* 2010; 12: 167-177

4.5 （介入研究）対象者に対する介入打ち切り基準

研究担当医師は以下の理由で研究継続が不可能と判断した場合には、研究薬の投与を中止し、中止・脱落の日付・時期、中止・脱落の理由、経過をカルテならびに症例報告書に明記するとともに、中止・脱落時点で必要な検査を行い有効性・安全性の評価を行う。

1. 有害事象の発現により、担当医師が投与を中止すべきと判断した場合
2. 過度の低血糖が認められかつ担当医師が中止すべきと判断した場合
3. 糖尿病の悪化（HbA1c12％以上）が認められかつ担当医師が中止すべきと判断した場合
4. 研究期間中ASTまたはALTが基準値上限の３倍を超えかつ継続した場合
5. 協力者が研究への参加の同意を撤回した場合
6. その他、担当医師が研究薬の中止が必要と判断した場合

注意１　：　いずれの場合も、中止の理由をカルテおよび症例報告書に記載する。

注意２　：　研究薬投与開始後に同意の撤回があった場合は、研究薬の効果不十分あるいは有害事象によるものか、あるいは偶発的事象（転居など）によるものかをできるだけ明らかにし、有効性・安全性評価の対象となる症例としての採否の参考となるように記録する。

4.6　（介入研究）コンプライアンスの確認方法

外来受診時に、主治医がコンプライアンスを確認する。コンプライアンスの確認は、あらかじめ評価の基準を定めて行う（100%服用、75%以上100%未満、50%以上75%未満、50%未満など）。

5　追跡・打ち切り

5.1 研究期間

2010年12月から2013年3月　（登録締切日　2012年9月）

5.2 （介入研究、前向き観察研究）追跡不能例に対する対処

脱落例とし、有効性評価には含めず、安全性に関する項目のみ、評価を行う。追跡不能例を最小限にするため、研究補助員からの定期的な電話連絡等を実施する。

6　(介入研究) 研究の中止

6.1 研究の中止基準

本研究の対象者に、重篤な有害事象が発生した場合には、試験薬に起因するものであるか否かを、実施責任者、部門長、共同研究機関の代表者で検討する。試験薬に起因すると判断された場合には、本研究の中止を検討する。

6.2 中止基準の確定法

自験例に重篤な有害事象が発生した場合には、直ちに倫理委員会、院内安全対策委員会へ報告し、迅速に必要な対応を行う。

自験例以外では、学会報告、その他あらゆる情報源より情報を収集し、倫理委員会との協議の上、決定する
