# Supplementary material for: Efficacy and Safety of Sitagliptin Added to Insulin in Japanese Patients with Type 2 Diabetes: The EDIT Randomized Trial
Source: PLoS One. 2015 Mar 27;10(3):e0121988. doi: 10.1371/journal.pone.0121988 (PMC4376939; doi:10.1371/journal.pone.0121988)
Supplement: S2 Protocol — (DOCX) [file pone.0121988.s007.docx]

Study protocol

1 Aims

To investigate the efficacy and the safety of DPP-4 inhibitor in type 2 diabetes mellitus inadequately controlled on intensive insulin therapy.

2 Study design

Prospective, open, randomized and comparative trial

0M 2W 1M 2M 3M 4M 5M 6M

Group B：　insulin therapy （n = 25）

Entry

Lab

Lab

Lab

Lab

Observation

-1M

(Insulin therapy）

Meal test

Lab

Lab

Lab

Group A：　insulin + sitagliptin therapy （n = 25）

Meal test

1) Observation period (more than 1 month)

Patients with suboptimal glycemic control (HbA1c ≥6.9%; NGSP value) despite more than twice daily insulin injections are enrolled in the study and randomized to group A: insulin + sitagliptin and group B: insulin.

2) Study period (6 months)

Group A: Start sitagliptin 50 mg daily. If initial HbA1c <7.9%, insulin dose is reduced by 20%. Then the dose of insulin is adjusted according to the JDS guideline. If glycemic goal is not achieved, sitagliptin is increased to 100 mg daily after week 12.

Group B: Continue insulin therapy. Insulin dose is adjusted according to the JDS guideline.

If there is hypoglycemia, the attending physician may reduce insulin dose.

Concomitant medication

In group A, insulin secretagogues such as sulfonylurea and glinide is discontinued at week 0. The other medication is not changed during the study.

Randomization

N = 25 in each group is planned. After observation period, the patients is randomized based on minimization adjusting for HbA1c, sex, age, BMI and number of insulin injection.

Deviation: Due to a small sample size of the study and the shortage of the funding, we adopted randomization based on the computer-created table of random numbers instead of minimization.

3 Outcomes

3.1 Outcomes

Primary outcome

- Amount and rate of change in HbA1c after 6 months

Secondary outcomes

(1) Amount and rate of change of the following items and these AUC during meal test:

plasma glucose

CPR

intact proinsulin

glucagon

FFA

Active GLP-1

Total GIP

TG

Deviation: total GLP-1 was also measured.

(2) Amount and rate of change in the following items after 6 months of treatment:

Waist circumference

daily profile of capillary glucose by SMBG

Patients QOL questionnaire

Bio-markers for oxidant stress (8-isoprostane, 8-OH-dG)]

(3) Amount and rate of change in the following items after 1, 2, 3, 4, 5, 6 months of treatment:

Blood pressure

Body weight

HbA1c

GA

1,5-AG

CPR

amount and frequency of insulin treatment

Rate of hypoglycemic episodes

(4) Exploring analysis

Stratified analysis based on age, sex, HbA1c, BMI, insulin treatment.

(5) Safety

4 Study participants

4.1 Study population

Outpatients with type 2 diabetes in Keio University Hospital, Tokyo, Japan.

4.2 Inclusion criteria

Patients with type 2 diabetes who are suboptimally controlled (HbA1c ≥6.9%) despite more than twice daily insulin injections and:

- Patients whose oral hypoglycemic agents are not changed past 2 months
- Age 20 years or older
- Male and female
- Outpatient
- Informed consent obtained

4.3 Exclusion criteria

- Patients with type 1 diabetes
- Patients with moderate to severe renal impairment (male sCr ≥1.5 mg/dl, female sCr ≥1.3 mg/dl or eGFR <30 ml/min)
- Patients with severe liver impairment
- Patients who are disqualified from the study by investigator for any reasons

4.4 Sample calculation

Based on the prior study1), reduction in HbA1c was estimated -0.7 ± 0.7% and -0.1% ± 0.7% in group A and group B respectively. Minimal sample needed to test a statistical significance with α = 0.05, 1- β = 0.80 is 23 in each group. Taking into consideration of dropout, N = 25 in each group was planned.

1) T Vilsvoll et al. *Diabetecs Obesity and Metabolism* 2010; 12: 167-177

4.5 Discontinuation criteria of intervention

(1) The attending physician judges to stop the intervention due to adverse event

(2) Severe hypoglycemia

(3) Worsening of glycemia (HbA1c ≥12%)

(4) Increase in AST or ALT over the three times of reference value

(5) Withdrawal of the consent

(6) Anytime the attending physician judges to stop the intervention

4.6 Compliance

The attending physician confirms the medication adherence at every visit.

5 Follow-up

5.1 Study period

2010/12-2013/3 (enrollment -2012/9)

Deviation: Enrollment period was extended to 2013/3 because the study participants did not reach 50 in 2012/9.

5.2 Loss of follow up

Loss-of-follow up is not included in efficacy evaluation but included in safety evaluation.

6 Discontinuation of the study

6.1 Criteria of discontinuation

If severe adverse event occurs during the study, the investigators consider the discontinuation of the study.

6.2 Decision of discontinuation

Severe adverse event is reported to the ethical and safety committees and discussed on discontinuation of the study.
